# Estimating the asbestos-related lung cancer burden from mesothelioma mortality

**DOI:** 10.1038/bjc.2011.563

**Published:** 2012-01-10

**Authors:** V McCormack, J Peto, G Byrnes, K Straif, P Boffetta

**Affiliations:** 1 Section of Environment and Radiation, International Agency for Research on Cancer, 150 cours Albert Thomas, Lyon 69008, France; 2 Faculty of Epidemiology and Population Health, Department of Epidemiology, London School of Hygiene & Tropical Medicine, Keppel Street, London WC1E 7HT, UK; 3 Biostatistics Group, Section of Genetics, International Agency for Research on Cancer, 150 cours Albert Thomas, Lyon 69008, France; 4 Section of IARC Monographs, International Agency for Research on Cancer, 150 cours Albert Thomas, Lyon 69008, France; 5 Institute for Translational Epidemiology and Tisch Cancer Institute, Mount Sinai School of Medicine, One Gustave L. Levy Place, New York, NY 10029-6574, USA; 6 International Prevention Research Institute, Lyon, France

**Keywords:** asbestos, lung cancer, mesothelioma, chrysotile

## Abstract

**Background:**

Quantifying the asbestos-related lung cancer burden is difficult in the presence of this disease's multiple causes. We explore two methods to estimate this burden using mesothelioma deaths as a proxy for asbestos exposure.

**Methods:**

From the follow-up of 55 asbestos cohorts, we estimated ratios of (i) absolute number of asbestos-related lung cancers to mesothelioma deaths; (ii) excess lung cancer relative risk (%) to mesothelioma mortality per 1000 non-asbestos-related deaths.

**Results:**

Ratios varied by asbestos type; there were a mean 0.7 (95% confidence interval 0.5, 1.0) asbestos-related lung cancers per mesothelioma death in crocidolite cohorts (*n*=6 estimates), 6.1 (3.6, 10.5) in chrysotile (*n*=16), 4.0 (2.8, 5.9) in amosite (*n*=4) and 1.9 (1.4, 2.6) in mixed asbestos fibre cohorts (*n*=31). In a population with 2 mesothelioma deaths per 1000 deaths at ages 40–84 years (e.g., US men), the estimated lung cancer population attributable fraction due to mixed asbestos was estimated to be 4.0%.

**Conclusion:**

All types of asbestos fibres kill at least twice as many people through lung cancer than through mesothelioma, except for crocidolite. For chrysotile, widely consumed today, asbestos-related lung cancers cannot be robustly estimated from few mesothelioma deaths and the latter cannot be used to infer no excess risk of lung or other cancers.

Asbestos is an established human carcinogen, the major effects being on cancers of the lung and mesothelioma of the pleura and peritoneum (IARC, 1987). Inhalation of asbestos fibres predominantly occurs during occupational exposure including in mines, construction, shipyards, railway and textile industries. Restrictions or bans on the use of certain asbestos fibres were introduced in the United States and Europe from the 1980s onwards, but latency periods of several decades mean that the mesothelioma epidemic is yet to peak. In 2000, an estimated 43 000 malignant mesotheliomas worldwide were due to occupational exposures (Driscoll *et al*, 2005), the largest absolute burdens being in the United States, Australia, Japan, New Zealand and Western Europe (Peto *et al*, 1999). Global asbestos (chrysotile) consumption was 2.3 million tonnes in 2003, predominantly in the lower income countries (Virta, 2006). The asbestos-related lung cancer (ARLC) burden is more difficult to quantify as most lung cancers are not attributable to asbestos, unlike for mesothelioma (Bianchi and Bianchi, 2007). ARLCs occur on top of varying lung cancer incidence rates and they are not clinically distinguishable from those not caused by asbestos. Furthermore, reliable population-based data on asbestos exposure (by fibre type, length, age at exposure, intensity) and associated relative risks are not known precisely, preventing the calculation of population attributable fractions (PAFs).

We present two methods to estimate the relationship between ARLC deaths and mesothelioma deaths, using the latter as a proxy for asbestos exposure. The first is the ratio of absolute excess lung cancer deaths to mesothelioma deaths. However with a synergistic interaction between asbestos and smoking on lung cancer, the ARLC burden will be particularly large in settings where smoking rates are high, thus a more appropriate ratio may be a relative effect on lung cancer to the absolute effect on mesothelioma. Using published cohorts of asbestos-exposed workers, we summarise these two ratios by asbestos type to gauge the magnitude of the population-level ARLC burden in different countries.

## Materials and Methods

### Ratio estimates

We estimated the ratio of asbestos-related lung cancers to mesothelioma deaths from asbestos cohorts. Two ratios were calculated for each cohort. Method 1: the absolute ratio (*R*_1_) of the absolute number of ARLC deaths to mesothelioma deaths is estimated by *R*_1_=(*O*_LC_−*E*_LC_)/*O*_M_, where *O* and *E* refer to the observed deaths and expected deaths in the absence of asbestos, with subscripts LC and M referring to lung cancer and mesothelioma, respectively. *E*_LC_ was estimated as *O*_LC_/SMR, the SMR being the standardised mortality ratio for lung cancer, which was usually provided. Method 2: the proportional ratio (*R*_2_) is the ratio of the excess lung cancer percentage for every mesothelioma death in 1000 non-asbestos-related deaths, *R*_2_=[100 × (*O*_LC_−*E*_LC_)/*E*_LC_]/[1000 × *O*_M_/*E*_all_], where *E*_all_ refers to expected deaths from all causes in the absence of asbestos, estimated as observed deaths minus (excess lung cancers+mesotheliomas) (=*O*_all_−(*O*_LC_−*E*_LC_)−*O*_M_). We assumed no expected mesothelioma deaths in the absence of asbestos. Note *R*_2_=*R*_1_ × *E*_all_/(10 × *E*_LC_).

Given a particular type of asbestos exposure, ratio *R*_1_ will only be constant in populations with similar background smoking and lung cancer incidence rates if there is a proportional increase in lung cancer risk associated with exposure (i.e., constant relative risk). As lung cancer incidence rates vary between countries and over time, the ratio of the relative effect on lung cancer to the absolute effect on the mesothelioma mortality rate may be more constant. We attempt to estimate this in ratio *R*_2_. Its numerator is the excess relative risk percentage. The denominator would ideally be an age-standardised mesothelioma mortality rate, but as this rate was rarely reported in included cohorts, the number of mesothelioma deaths per 1000 non-asbestos-related deaths (*E*_all_) was used as a correlate. Thus *R*_2_ is the excess lung cancer relative risk (expressed as a percentage) for every mesothelioma death in 1000 non-asbestos-related deaths.

### Included studies and data extraction

Asbestos cohorts were identified via a PubMed search, last updated on 1 September 2011, of all articles containing the MeSH terms ‘Neoplasms’, ‘Cohort Studies’ and ‘asbestos’, yielding 425 results. Reference lists of retrieved articles and previous relevant reviews were also checked for omissions (Hodgson and Darnton, 2000; Boffetta, 2007). For the British Asbestos Survey, data were extracted from an online report (Harding, 2010). Eligible studies included were those with (i) at least 100 subjects, (ii) certain asbestos exposure (not just probable such as in environmental settings) and (iii) observed and expected (based on country-level rates or an unexposed cohort) mesothelioma and lung cancer mortality or incidence for the same follow-up period. Cohorts consisting entirely of asbestosis or pleural disease patients were not included. For cohorts with multiple publications, the most informative recent one was included. From each included publication we extracted information on cohort characteristics, asbestos fibre type (crocidolite/chrysotile and crocidolite/pure or predominantly chrysotile/amphiboles including amosite/mixed or unspecified), industry, cause of death ascertainment, whether lung cancer SMRs were adjusted for smoking (or whether smoking prevalence was comparable with that of the reference population), total number of deaths and observed and expected deaths from lung cancer and mesothelioma. Incidence data were used if mortality data were not available (not contributing to *R*_2_). Expected deaths were usually estimated by authors from population-level age-sex-period-specific mortality rates. For the Great Britain Asbestos Survey, excess lung cancers were calculated from proportional mortality ratios rather than SMRs in order to minimise confounding by smoking (Harding, 2010). Where appropriate, mortality was based on best evidence rather than death certificate only. In some instances only pleural mesotheliomas were recorded (indicated in tables).

As our aim was to summarise the overall association between the ARLC and mesothelioma mortality to reflect estimates of the average association effect, we did not carry out a meta-regression of all possible factors that might affect an individual ratio (other than asbestos type and confounding by smoking). This average association is useful because a mesothelioma burden at the population level arises from differing exposure conditions, as reflected in those from the various cohorts included.

### Statistical methods

Extracted data were used to calculate *R*_1_ and *R*_2_ as defined. Estimates of *R*_1_ were plotted by asbestos type, with the variance of log(*R*_1_) estimated as (1/*E*_LC_+1/*E*_M_) and for *R*_2_ as (1/*E*_LC_+1/*O*_M_+1/*O*_all_). Summary values of *R*_1_ and *R*_2_ and of SMRs for lung cancers were obtained from a random-effects meta-analysis implemented in STATA version 11. Meta-regression models were used to examine whether the ratio estimates systematically differed between studies, where smoking was controlled for or not. To evaluate which of the ratios *R*_1_ and *R*_2_ was most appropriate, we assessed which one explained a greater proportion of the variation when regressing the numerators on the denominators of the respective ratios, weighted by the inverse of the appropriate variances given above.

To apply the ratio estimates to external larger populations, ARLCs were calculated using method 1, as *R*_1_ times the number of mesothelioma deaths (*R*_1_ × *O*_M_), or using method 2, PAFs for lung cancer due to asbestos were calculated as (1+0.1 × *O*_all_/(*R*_2_ × *O*_M_))^−1^. Using these results, we calculated PAF ranges for men aged 40–84 years during years 2001–2005, using the WHO mortality database for selected countries with mortality data coded to ICD-10 (WHOSIS Mortality Database ICD-10, 2009).

Population attributable fraction estimates were based firstly on ratio estimates as provided in Tables 1 and 2, and thereafter on ratio estimates corrected for smoking if smoking had not been accounted for. The correction applied was to reduce ratio estimates by 25% if smoking had not been taken into account, which corresponds to the degree of positive confounding in the lung cancer SMR, resulting from a smoking prevalence of 70% in the asbestos cohort compared with 50% in the general population and a smoking relative risk of 10. This is likely to be an exaggerated correction and thus possibly underestimates ratio and PAF estimates.

## Results

### Ratio estimates

Fifty five publications were included, from which 68 risk estimates were reported, 6 for crocidolite, 8 for crocidolite and chrysotile, 14 for predominantly chrysotile, 4 for amosite and 32 to mixed or unspecified asbestos fibres (Table 1, with an extended version in online Supplementary Table 1). In total, follow-up included over 67 194 deaths, of which 1963 were from lung cancer and 1962 from mesothelioma. Most studies were conducted in North America or Europe, with only seven in other regions (Japan, Australia, China and South Africa). The two largest cohorts were the insulation workers’ union of the United States and Canada (*n*=17 000), and the Great Britain Asbestos Survey of over 98 000 registered asbestos workers (Selikoff and Seidman, 1991; Harding, 2010). Lung cancer SMRs for 78% of cohorts were not adjusted for smoking, other than in a few studies where internal analyses comparing exposed to unexposed subjects adjusted for smoking and in certain cohorts smoking prevalence of workers was noted to be comparable (or not) to that of the general population. We used the proportional mortality ratio in the British Asbestos survey in an attempt to get closer to a smoking-adjusted SMR (estimates 66 and 67).

**Figure 1:**
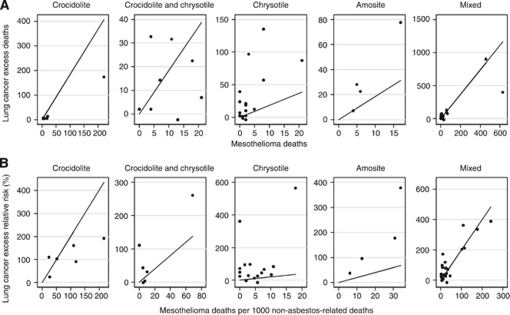
(**A**) Ratio 1 components, absolute excess lung cancer excess deaths *vs* mesothelioma deaths, by asbestos type. (**B**) Ratio 2 components, lung cancer excess relative risk (%) *vs* mesothelioma deaths per 1000 non-asbestos-related deaths, by asbestos type. Note that graphs are not to the same scale. The reference line on each sub-graph corresponds to the same ratio estimate (average values for mixed fibres from Table 3); thus, the distribution of points about this line can be compared across subgraphs.

Figure 1 plots absolute and relative lung cancer excess *vs* mesotheliomas, that is, the numerator and denominators of ratios *R*_1_ and *R*_2_, by asbestos type. Note that axes for each subplot are on different scales, particularly for mesothelioma because of greatly varying mesothelioma-producing potential of the different fibres. The line on each plot corresponds to two excess lung cancer deaths per mesothelioma death, that is, this is the same ratio across graphs to aid comparison.

#### Crocidolite and crocidolite plus chrysotile

**Figure 2:**
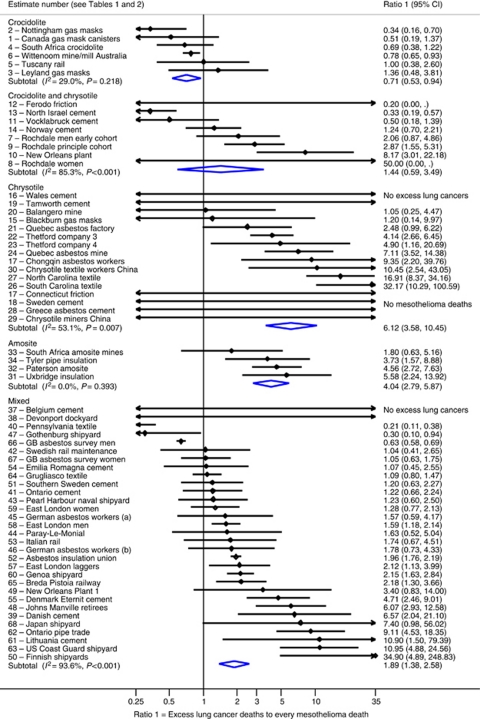
Ratio of asbestos-related lung cancers to mesothelioma deaths, study-specific and random effects combined estimates by asbestos fibre type. Estimates of the ratio of excess lung cancer deaths to every mesothelioma death (*R*_1_) in each study, by asbestos fibre type. *I*^2^ represents the percentage of the variation in the study-specific estimates that are due to true heterogeneity rather than due to chance.

In the six crocidolite-exposed cohorts, workers experienced increased lung cancer mortality, of the order of a two-to three-fold increase (pooled SMR 2.0, Table 3), and increased mesothelioma risk, with mesothelioma deaths contributing to a median of 93.2 per thousand deaths (range 23–217). In all but two smaller cohorts, there were less or equivalent numbers of excess lung cancers compared with mesothelioma deaths, giving a combined *R*_1_ estimate of 0.71 ARLC deaths per mesothelioma death (Table 3, Figure 2). The combined estimate of ratio *R*_2_ suggests a 1.2% increase in lung cancer deaths (PAF=1.2%) associated with crocidolite for every mesothelioma death in 1000 deaths.

For cohorts with a mixture of chrysotile and some crocidolite, mesothelioma risk was also raised but to a lesser extent than for pure crocidolite (7.6 mesothelioma deaths per 1000 non-asbestos-related deaths). Lung cancer risk was increased (SMR 1.58), varying from no lung cancer excess in the Ferodo friction factory (estimate 12) to increases of over three-fold (Table 1 and Figure 2). These combined to give an overall *R*_1_ estimate of 1.44 excess lung cancers per mesothelioma death (Table 3).

#### Chrysotile

Chrysotile cohorts had a wider range of estimates, resulting from little correlation between excess lung cancers and mesotheliomas (Figures 1 and 2). Almost all SMRs for lung cancer were between 1 and 2, with two notable exceptions of two Chinese mines (estimates 25 and 29). In 14 of 16 estimates, the number of excess lung cancer deaths was more than the number of mesothelioma deaths (Figure 2); an average *R*_1_ of 6.1 excess lung cancers per mesothelioma death (Table 3). This much larger ratio, compared with that for crocidolite, arises mainly from a much smaller associated mesothelioma risk, including four cohorts that had no mesothelioma deaths (estimates 17, 18, 28 and 29, Table 1). The small denominator and wide range makes ratio estimates of excess lung cancers to mesotheliomas large, but imprecise for chrysotile.

#### Amphiboles

In the follow-up of four amosite cohorts, there was a marked correlation between lung cancer SMR and mesothelioma. The combined SMR for lung cancer was 2.5, slightly stronger than that for crocidolite and thus, with a mesothelioma risk approximately one-fifth of that for crocidolite (18 *vs* 93 mesotheliomas per 1000 deaths in amosite and crocidolite, respectively, Table 3), ratio *R*_1_ was much higher at 4 excess lung cancers per mesothelioma death (Table 3). Corresponding *R*_2_ estimates suggest there was between a 6% and 10% increase in lung cancer deaths for every mesothelioma death in 1000 deaths. The largest contributing study was the Paterson amosite factory in New Jersey (estimate 32), where both a large number of mesotheliomas (17 in 820 workers) and a very large excess lung cancer mortality were observed (SMR 4.8) (Seidman *et al*, 1986). In the only cohort with anthophyllite exposure (estimate 35), there were 12.5 excess lung cancers per mesothelioma death (Meurman *et al*, 1994).

#### Mixed asbestos types

For the majority of cohorts asbestos exposure was mixed and their ratio estimates lay between the corresponding values for pure asbestos fibres. The summary SMR for lung cancer was 1.77 (95% CI 1.44, 2.20), which was less than that for crocidolite or amosite, but larger than that for chrysotile. Mesothelioma deaths as a proportion of total mortality were half that for crocidolite, but higher than chrysotile or amosite (Table 3). There were between one and three excess lung cancers for every mesothelioma death in half of the cohorts, but the estimate in the two largest cohorts were very different, at 0.6 in men in the Great Britain Asbestos Survey and 2.0 in US and Canadian insulation workers (estimates 55 and 66, Table 1). For all mixed asbestos cohorts combined, there were 1.9 (95% CI 1.4, 2.6) excess lung cancer deaths for every mesothelioma death, although with large heterogeneity.

#### Comparison of the two ratio estimates

The percentage of the variance explained by regression of the contributing numerator on denominator was higher for *R*_1_ (>95%) than for *R*_2_ for crocidolite, crocidolite+chrysotile and for mixed asbestos cohorts, but was higher for *R*_2_ than *R*_1_ for chrysotile and amosite cohorts.

### Population attributable fractions in men

Table 4 provides, for 20 countries with large mesothelioma proportional mortality, PAFs (%) of male lung cancer deaths due to asbestos estimated using the crude and smoking-adjusted estimates of ratios *R*_1_ and *R*_2_ for exposure to mixed asbestos fibres, and smoking-adjusted ratios for crocidolite. The smoking adjustment (derived from 20% higher cohort smoking prevalence than the general population) is likely to underestimate ratios and PAFs, and can be considered a lower limit. In the countries listed, mesothelioma deaths ranged from 1 to 9 per 1000 deaths. If the ratio of ARLC to mesothelioma deaths were exactly 1, then mesothelioma deaths as a percentage of lung cancer deaths (ranging from 1% to 9% for these countries) would be the PAF (%) of lung cancer due to asbestos. For Australia, at the top of the table with the highest percentage of deaths from mesothelioma, using ratio estimates for crocidolite, we estimated that between 5.4% and 8.6% of lung cancer deaths in men are asbestos-related, based on *R*_1_ and *R*_2,_ respectively. Similarly in the United Kingdom, New Zealand, South Africa and the Netherlands, where crocidolite was used among other asbestos fibres, PAFs ranged between 4% and 7%. For South Africa calculations based on *R*_1_ estimates (PAF 6.7–8.4% for mixed fibres, 2.7% for crocidolite) are more suitable as total background mortality, which is incorporated into *R*_2_-based estimates, is particularly high in this country during the HIV era. Applying the ratios for mixed asbestos exposures, PAFs for other countries lie between 3% and 7% using both ratio methods of estimation. For the United States, the estimated PAF in men was estimated to be 3.2–4.0% for mixed asbestos (*R*_1_ estimates).

## Discussion

The ratios of ARLCs to mesotheliomas fall into three categories: (1) pure amphibole or mixed exposures causing high lung cancer risks and ARLC/mesothelioma ratios ranging from <1 for crocidolite, 1.4 to 2.5 for mixed types, to 4 or more for amosite and anthophyllite; (2) heavy prolonged chrysotile exposures causing high lung cancer risks and ARLC : mesothelioma ratios of the order of 10; and (3) shorter or less heavy chrysotile exposures causing lower lung cancer risks and little mesothelioma, between which there was little correlation and thus calculation of excess lung cancers from mesothelioma deaths after chrysotile exposure would not be reliable. Thus for all asbestos types other than crocidolite, the ARLC is larger than that for mesothelioma, but the low ARLC to mesothelioma ratio for crocidolite (0.71) does not imply that the lung cancer risk is small, only that it is slightly smaller than the high mesothelioma risk.

The ARLC : mesothelioma ratio in chrysotile cohorts may be dominated by two large errors. First, the lung cancer excess depends critically on the rates on which the SMR is based. Second, it seems likely that many of the mesotheliomas in such cohorts are actually due to amphibole exposure. These points are illustrated by the chrysotile miners and millers in Quebec (estimates 21–24). Their ARLC : mesothelioma ratio varied substantially, from 3.3 (22 mesotheliomas; 423 lung cancers, SMR 1.21) for cumulative exposures below 300 million particles per cubic foot (mpcf)-years to 31 (1 mesothelioma; 47 lung cancers, SMR 2.97) above 1000 mpcf-years. The lung cancer excess above 1000 mpcf-years is affected little by the choice of rates, but below 300 mpcf-years the lung cancer excess could be doubled or reduced to zero by a 20% change in expected rates. Furthermore, many mesotheliomas occurring in asbestos that is predominantly chrysotile may actually be due to other asbestos types. McDonald *et al* (1997) argued that the mesotheliomas among chrysotile miners and millers in Quebec were caused either by amphibole exposure elsewhere or by the tremolite that contaminated most Canadian chrysotile. Analysis of 22 lung samples from mesotheliomas in this cohort showed that 6 contained substantial amounts of crocidolite, and only 2 contained more chrysotile than tremolite despite their much heavier exposure to chrysotile. Tremolite, similarly to crocidolite and amosite, is an amphibole that is cleared less rapidly from the lung than chrysotile. The vermiculite miners in Montana were included on this basis as vermiculite contains tremolite. Nevertheless, irrespective of the contribution of tremolite, it seems that prolonged heavy exposure to chrysotile causes a much larger excess of lung cancer than of mesothelioma.

### Heterogeneity in ratio estimates within and between cohorts

Neither ratio *R*_1_ or *R*_2_ appeared to be more constant across all cohorts. Although it was expected that *R*_2_ would more appropriately account for differential background lung cancer incidence rates, and thus be a more constant ratio, its denominator – ideally an age-adjusted mesothelioma mortality rate could not be calculated – was mesothelioma proportional mortality and thus was additionally affected by differential background mortality rates. Nonetheless, PAF estimates using either ratio did not differ hugely.

We have considered the effect of fibre type on ratios; however, several other factors, discussed below, will affect within and between study ratio estimates. Stratifying by each factor could not be conducted owing to lack of data. The summary ratios provided here thus better characterise the overall ARLC–mesothelioma relationship across exposure circumstances and over a long period of time, and do not serve to precisely quantify lung cancer excess in a short time period.

Dose–response relationship: Most formal risk assessment analyses assume that the relative risk for lung cancer is increased by each brief period of asbestos exposure in proportion to dose (intensity times duration), whereas the increase in the absolute mesothelioma rate is proportional to the dose weighted by the square or cube of latency. Such models imply that prolonged exposure beginning in middle age will cause a ratio of ARLC to mesothelioma more than 10 times greater than brief exposure at age 20 years (Peto *et al*, 1985). The ratio is thus age-dependent and affected by differences in the length of follow-up between studies. Exposure intensity also varies between and within cohorts, for example, if factory ventilation systems improve.
Death certificate reporting bias: Inaccurate recording of deaths may lead to possible overestimation of ratios, as shown by an autopsy-death certificate comparison in which false-negative mesotheliomas were likely to be recorded as lung cancers (Delendi *et al*, 1991). Special efforts to capture all mesothelioma deaths from medical records and mesothelioma registries would have reduced this bias here and mesothelioma-reporting accuracy should improve over time with the use of ICD-10.
Overall mortality rates: Although the use of a relative effect on lung cancer has been captured in *R*_2_, its denominator would ideally be an age-standardised mesothelioma mortality rate. Instead the mesotheliomas per 1000 deaths are affected by changes in the overall risk of death. Mesothelioma proportional mortality will be higher in cohorts with lower mortality.
Confounding/effect modification by smoking and other lung carcinogens: Ratio estimates hinge on the assumption that SMRs for lung cancer are unbiased. Several cohorts specifically adjusted for smoking (estimates 11, 25, 29, 30, 68) and in some others estimates of smoking prevalence in subsets suggest that they were similar to that of the general population (estimates 43, 50, 51). Use of the PMR (1.28) rather than the SMR (1.89) for lung cancer was intended to reduce confounding by smoking (estimates 66, 67; Harding, 2010). The extent to which bias may be present in other cohorts depends on the social-class smoking gradient. Two recent cohorts commented on a higher smoking prevalence (estimates 61, 62) than in the general population, which may explain their high ratios. In addition, asbestos has a stronger effect on lung cancer in smokers than non-smokers, whereas smoking has little or no effect on mesothelioma. Thus, higher baseline lung cancer mortality rates may explain the higher ratios observed in United States and the European cohorts than in Israel (estimate 13, with a low lung cancer mortality rate; Tulchinsky *et al*, 1999). The great majority of cohorts included here were from Europe, the United States and Canada, where asbestos exposure occurred predominantly during the 1940s to 1970s when smoking prevalence was high. Estimates for Ontario pipe traders and two cohorts of shipyard workers (cohorts 50 and 63) may be overestimated if these workers also had exposure to other lung carcinogens, such as lead, chromium, cadmium and diesel exhaust.

### Population-level inferences

For countries with the highest percent deaths from mesothelioma in men, estimates of the lung cancer PAF due to asbestos were between 3% and 8% for 2001–2005. In almost all of these countries, lung cancer made up 20–30% of cancer deaths, thus even 3–8% of this is a considerable contribution to the most common cause of cancer mortality in men in these settings. A few previous lung cancer-asbestos PAF estimates have been made. For the United Kingdom, the British Asbestos Survey included here had an *R*_1_ value of 0.6, much lower than the other mixed asbestos cohorts. Darnton *et al* (2006) obtained a similar estimate (*R*_1_ between 0.7 and 1) from a population-based analysis of the relationship between lung cancer and mesothelioma PMRs in men in 131 occupational groups, adjusting for smoking, and a PAF of 2% to 3%. This is approximately half of the estimate found here (4.9% to 7.7% using crocidolite ratios, from Table 3), and the difference may be because their estimate was for a longer earlier period (1980–2000) than the current estimates (for 2001–2005), during which time mesotheliomas have increased as a percentage of lung cancer deaths (from 3.8% to 8.1%). However, as amosite was a major cause of mesothelioma in the United Kingdom (Rake *et al*, 2009), the lung cancer excess in the United Kingdom would be expected to be even higher. For Italy, Marinaccio *et al* (2008) used a model-based approach to estimate the ARLC burden in men in 1980–2001 and found PAFs for lung cancer mortality of between 1.6% and 3.7%, that is, a ratio (*R*_1_) of between 0.68 and 1.37 excess lung cancers per mesothelioma. These estimates are similar to those of Darnton *et al* (2006), and also suggest a slightly lower ratio of ARLCs to mesotheliomas than that based on our estimated ratios from mixed asbestos cohorts. In the United States, where amphiboles constituted a much smaller proportion of asbestos consumption than in Europe, estimates of the male lung cancer PAF for asbestos were 6% to 8% in the 1990s (Nicholson *et al*, 1982; Morabia *et al*, 1992), slightly higher than the estimate of 4.0–5.0% here. In Finland, where anthophyllite was mined extensively, 2.7 mesothelioma deaths per 1000 would lead to a PAF for lung cancer of over 11% (using *R*_2_=4.4), consistent with Nurminen and Karjalainen (2001) estimate of 14%. Attempts to estimate ARLCs at a population level should be made with additional caveats. The majority of asbestos exposures at the population level may be of lower intensity and for longer durations, and the largest exposed groups may not be represented in asbestos cohorts, for example, construction workers and carpenters in the United Kingdom (Rake *et al*, 2009).

The asbestos-related cancer burden today is predominantly in Europe, North America, Australia, Japan, South Africa and parts of South America. However, the highest current consumption and exposure, and thus future burden, is in China, Russia, India, Kazakhstan, Ukraine, Thailand, Brazil and Iran. Exposure today is predominantly to chrysotile, either in a pure form or naturally contaminated by tremolite. The ratios presented demonstrate that the mesothelioma-producing potential of chrysotile is low and thus the number of mesothelioma deaths will be too unstable to be used to estimate the lung cancers caused by it. Critically, few mesotheliomas associated with chrysotile exposure cannot be used to imply a small asbestos-related lung cancer burden. The major effect of the continuing use of this carcinogen will be on lung cancer. High smoking rates in men (e.g., 70% in Russia, 61% in China (World Health Organization, 2008)) in these countries will amplify the associated lung cancer burden. There is thus an urgent need for limiting exposure through strict regulation of asbestos use, and encouragement of smoking cessation to reduce mortality among formerly exposed workers.

## Figures and Tables

**Table 1:** Crocidolite, chrysotile and amosite asbestos cohorts providing estimates of lung cancer and mesothelioma mortality, by asbestos type

**No.**	**Cohort description (reference)**	***N***	**% Male**	**Smoking adjustment**	**Total deaths**	**Lung cancer deaths (*O*_LC_)**	**Lung cancer SMR**	**Mesothelioma deaths (*O*_M_)**	**Ratio *R*_1_**	**Ratio *R*_2_**
*Crocidolite*										
1	Canadian gas mask canisters (McDonald and McDonald, 1978)	199	NK	N	55	7	2.92	9	0.5	0.9
2	Nottingham gas masks, UK (Jones *et al*, 1980)	951	0	N	166	12	1.90	17	0.3	0.8
3	Leyland gas masks UK. Crocidolite, some chrysotile (Acheson *et al*, 1982)	757	0	N	219	13	2.10	5	1.4	4.6
4	South African crocidolite mines (Sluis-Cremer *et al*, 1992)	3430	100	N	423	27	2.03	20	0.7	2.0
5	Tuscany rail construction Italy (Battista *et al*, 1999)	734	100	N	199	26	1.24	5	1.0	0.9
6	Wittenoom mine/mill, Australia (Musk *et al*, 2008)	6943	100	C	2408	281	2.60	222	0.8	1.5
										
*Chrysotile and crocidolite*										
7	Rochdale textile workers, UK. Chrysotile, 5% crocidolite during 1932–1968 (Peto *et al*, 1985): men, 20+ years employment starting <1933	145	100	N	123	20	3.61	7	2.1	3.7
8	As for 7: Rochdale women, 10+ years employment after 1933	283	0	N	49	4	2.11	0	No meso.	
9	As for 7: Rochdale principal cohort, men first employed >1933	3211	100	N	1113	132	1.31	11	2.9	3.1
10	New Orleans cement Plant 2, USA (Hughes *et al*, 1987)	3594	100	C	874	107	1.44	4	8.2	9.2
11	Vocklabruck cement workers, Austria (Neuberger and Kundi, 1990)	2816	Both	A	540	49	1.04	4	0.5	0.6
12	Ferodo friction factory, UK. Predominantly chrysotile, crocidolite use for a short period (Berry, 1994)	13 450	68	N	2577	241	0.99	13	−0.2	−0.2
13	North Israel cement workers, 90% chrysotile, 10% crocidolite (Tulchinsky *et al*, 1999)	3057	100	N	*—*	34	1.26	21	0.3	*—*
14	Norwegian asbestos cement factory. Chrysotile, 8% amphiboles (Ulvestad *et al*, 2002)	545	100	N	*—*	33	3.1	18^a^	1.2	*—*
										
*Chrysotile (pure or predominantly)*										
15	Blackburn gas masks, UK. Pure chrysotile (Acheson *et al*, 1982)	570	0	N	177	6	1.25	1	1.2	4.4
16	Wales cement workers. After 1936, chrysotile only. Before 1936, some crocidolite used (Thomas *et al*, 1982)	1970	100	N	351	22	0.85	2^b^	−1.9	−2.6
17	Connecticut friction industry, USA. Pure chrysotile^c^ (McDonald *et al*, 1984)	3531	100	N	803	73	1.49	0	No meso.	
18	Swedish cement workers. Chrysotile predominantly, <1% amosite/crocidolite during short periods (Ohlson and Hogstedt, 1985)	1176	100	C	220	11	1.22	0	No meso.	
19	Tamworth cement workers, UK. Chrysotile, with amosite used during 4 months out of 42 years (Gardner *et al*, 1986)	2167	70	N	486	41	0.97	1	−1.4	−1.6
20	Balangero mine, Italy. Pure chrysotile (Piolatto *et al*, 1990)	1058	100	N	427	22	1.11	2^a^	1.1	2.2
21	Quebec, Canada: Quebec asbestos factory, chrysotile (Liddell *et al*, 1997)	792	100	N	508	49	1.34	5	2.5	3.6
22	As for 21: Thetford company 3, chrysotile and tremolite	4732	100	N	3080	280	1.45	21	4.1	7.2
23	As for 21: Thetford company 4, chrysotile+tremolite	368	100	N	267	25	1.65	2	4.9	9.5
24	As for 21: Quebec asbestos mine/mill, chrysotile	4503	100	N	2924	253	1.29	8	7.1	11.8
25	Chongqin asbestos workers, China. Pure chrysotile, tremolite content uncertain (Yano *et al*, 2001)	515	100	A	132	22	6.64	2	9.3	31.4
26	South Carolina textile workers, USA. Chrysotile, <0.01% crocidolite (Hein *et al*, 2007)	3072	59	C	1961	198	1.95	3	32.2	58.9
27	North Carolina textile plants, USA. Predominantly chrysotile, with some amosite in plant 3 (Loomis *et al*, 2009)	5770	69	N	2583	277	1.96	8^d^	16.9	29.1
28	Greece asbestos cement factory. Chrysotile with 0.5% amphibole contamination (Sichletidis *et al*, 2009)	317	100	N	52	16	1.71	0	No meso.	
29	Chrysotile miners, China (Wang X *et al*, 2011)	1080	100	A	343	50	4.61	0	No meso.	
30	Chrysotile textile workers, China (Wang XR *et al*, 2011). Medium- and high-exposed groups compared with low-exposed group	461	100	A	207	46	1.83	2	10.4	7.6
										
*Amosite*										
31	Uxbridge amosite insulation, UK (Acheson *et al*, 1984)	4820	100	N	422	57	1.96	5	5.6	7.5
32	Paterson amosite asbestos factory, NJ, USA (Seidman *et al*, 1986)	820	100	N	593	98	4.78	17	4.6	11.1
33	South African amosite mines (Sluis-Cremer *et al*, 1992)	3212	100	N	648	26	1.38	4	1.8	6.1
34	Tyler pipe insulation, amosite, TX, USA (Levin *et al*, 1998)	1130	100	N	222	35	2.78	6	3.7	5.7
										
*Anthophyllite and vermiculite*										
35	Finnish anthophyllite mines, Paakkila, Maljasalmi (Meurman *et al*, 1994)	903	82	N	*—*	77	2.86	4	12.5	*—*
36	Vermiculite mine, Libby Montana, USA (contains tremolite) (Sullivan, 2007)	1672	96	N	711	89	1.69	6	6.1	7.7

Abbreviations: A=adjusted; C=smoking prevalence in cohort comparable to the general population; N=none or not mentioned; SMR=standardised mortality ratio.

a Pleural mesothelioma only.

b The two mesotheliomas were in men who were employed before 1936 when exposure to crocidolite was likely.

c Deaths 20 years after first employment only.

d Four cancers of pleura and four mesothelioma (the latter only available from 1999 with ICD10). The four mesotheliomas were in workers who had been employed in plant 4, where there was no record of amphibole use. Three pleural cancers were in plant 3, but these workers had not worked in the insulation section of plant 3, where amosite was used.

**Table 2:** Cohorts with exposure to mixed asbestos fibres: lung cancer and mesothelioma mortality and ratio estimates

**No.**	**Cohort description (reference)**	***N***	**% Male**	**Smoking adjustment**	**Total deaths**	**Lung cancer deaths (*O*_LC_)**	**Lung cancer SMR**	**Mesothelioma deaths (*O*_M_)**	**Ratio *R*_1_**	**Ratio *R*_2_**
*Mixed (or unspecified) asbestos types*										
37	Belgian asbestos-cement factory (Lacquet *et al*, 1980)	1973	100	N	201	17	0.99	1	−0.2	−0.3
38	Devonport dockyard, UK (Rossiter and Coles, 1980)	6292	100	N	1043	84	0.84	31	−0.5	−0.5
39	Danish asbestos-cement factory (Clemmesen and Hjalgrim-Jensen, 1981)	6372	100	N	*—*	47	1.72	3	6.6	*—*
40	Pennsylvania textile, USA. Mostly chrysotile, some amosite, small amounts crocidolite (McDonald *et al*, 1983)	4137	100	N	1392	57	1.05	13	0.2	0.5
41	Ontario cement workers, Canada (Finkelstein, 1984)	535	100	N	108	26	4.89	17	1.2	1.6
42	Swedish rail maintenance (Ohlson *et al*, 1984)	3297	100	N	925	37	1.16	5	1.0	3.0
43	Pearl Harbour naval shipyard, USA (Kolonel *et al*, 1985)	5191	100	C	668	122	1.09	8	1.2	0.7
44	Paray-Le-Monial cement factory, France (Alies-Patin and Valleron, 1985)	1506	100	N	206	12	2.18	4	1.6	5.8
	German asbestos workers (Woitowitz *et al*, 1986)		NS	N						
45	(a) Exposed before 1972 only	655			71	12	4.62	6	1.6	3.6
46	(b) Exposed before and after 1972	3070			185	26	1.70	6	1.8	2.0
47	Gothenburg shipyard, Sweden (Sanden and Jarvholm, 1987)	3787	100	N	*—*	11	1.12	4	0.3	*—*
48	Mixed industry, Johns Manville retirees, USA (Enterline *et al*, 1987)	1074	100	N	944	77	2.71	8	6.1	20.2
49	Cement, New Orleans Plant 1, USA. Primarily chrysotile, irregular use of amosite and crocidolite (Hughes *et al*, 1987)	1898	100	C	477	47	1.17	2	3.4	4.0
50	Finnish shipyard workers (Tola *et al*, 1988)	7775	100	C	*—*	227	1.18	1	34.9	*—*
51	Southern Sweden cement workers (Albin *et al*, 1990)	1465	100	N	592	35	1.80	13	1.2	3.5
52	Asbestos insulation union, USA–Canada (Selikoff and Seidman, 1991)	17 800	100	N	4951	1168	4.35	458	2.0	2.6
53	Italian rail carriage construction (Menegozzo *et al*, 1993)	1534	100	N	194	28	1.45	5	1.7	1.6
54	Emilia Romagna cement factory, Italy. Chrysotile and 5–50% crocidolite (Giaroli *et al*, 1994)	3341	100	N	274	33	1.24	6	1.1	1.1
55	Danish Eternit cement factory, 90% chrysotile, amosite and small amounts crocidolite (Raffn *et al*, 1996)	7996	100	C	1305	104	1.83	10	4.7	10.3
56	East London asbestos workers UK (Berry *et al*, 2000) of which:	5100		N	1237	233	3.03	100	1.6	2.0
57	As for 56: East London laggers	1400	100	N	*—*	38	3.67	13	2.1	*—*
58	As for 56: East London men	3000	100	N	*—*	157	2.55	60	1.6	*—*
59	As for 56: East London women	700	0	N	*—*	37	7.46	25	1.3	*—*
60	Genoa shipyard, Italy (Puntoni *et al*, 2001)	3984	100	C	2376	298	1.77	60	2.2	2.8
61	Lithuania cement, Naujoji Akmeme Lithuania (Smailyte *et al*, 2004)	2498	69	N	450	38	1.40	1	10.9	17.5
62	Ontario pipe trade, Canada (Finkelstein and Verma, 2004)	25 285	100	N	2876	393	1.23	8	9.1	8.0
63	US Coast Guard shipyard workers (Krstev *et al*, 2007)	4702	94	N	3331	314	1.26	6	11.0	14.4
64	Grugliasco textile Italy. Mixed asbestos with crocidolite (Pira *et al*, 2007)	1966	45	N	730	109	3.11	68	1.1	1.8
65	Breda Pistoia railway rolling factory, Italy (Gasparrini *et al*, 2008)	1146	100	N	1080	132	1.36	16	2.2	2.3
	Great Britain Asbestos Survey (Harding, 2010)									
66	Men	94 403	100	C	14 677	1802	1.28^a^	631	0.6	0.6
67	Women	4509	0		880	84	1.29^a^	18	1.1	1.4
68	Japanese shipyard (laggers and boiler repairers) (Tomioka *et al*, 2011)	249	100	C	158	15	1.97	1	7.4	14.5

Abbreviations: A=adjusted; C=smoking prevalence in cohort comparable to the general population; N=none or not mentioned; SMR=standardised mortality ratio.

a Proportional mortality ratio reported rather than SMR.

**Table 3:** Estimated ratios by asbestos fibre type for (i) absolute excess lung cancer deaths to mesothelioma deaths and (ii) excess lung cancer percentage to mesothelioma deaths per 1000 non-asbestos-related deaths

	**Lung cancer mortality SMR**		**Ratio *R*_1_ excess lung cancers per mesothelioma death**		**Ratio *R*_2_ excess lung cancer (%) for every mesothelioma death in 1000 non-asbestos-related deaths**	
**Asbestos type (no. of estimates)**	**Mean (95% CI)**	**Mesothelioma deaths per 1000 non-asbestos-related deaths**	**Median (IQR)**	**Mean (95% CI)**	**Median (IQR)**	**Mean (95% CI)**
Crocidolite (6)	2.04 (1.55, 2.69)	93.2	0.7 (0.5, 1.1)	U 0.7 (0.5, 1.0)	1.2 (0.9, 2.6)	U 1.4 (1.0, 2.1)
				SA 0.6 (0.3, 0.9)		SA 1.2 (0.8, 1.8)
Chrysotile and Crocidolite (8)	1.58 (1.19, 2.08)	7.6	1.7 (0.4, 6.8)	U 1.4 (0.6, 3.5)	3.4 (0.4, 9.4)	U 2.8 (1.0, 7.5)
				SA 1.2 (0.5, 2.9)		SA 2.4 (0.9, 6.3)
Chrysotile (16)	1.68 (1.39, 2.03)	4.1	8.2 (1.5, 11.0)	U 6.1 (3.6, 10.5)	9.1 (3.6, 10.3)	U 10.2 (5.5, 18.9)
				SA 4.9 (2.8, 8.7)		SA 8.3 (4.3, 15.9)
Amosite (4)	2.48 (1.42, 4.33)	18.6	4.1 (2.3, 5.3)	U 4.0 (2.8, 5.8)	6.8 (5.8, 10.2)	U 8.4 (5.9, 11.8)
				SA 3.0 (2.1, 4.4)		SA 6.3 (4.5, 8.8)
Mixed (31)	1.77 (1.44, 2.20)	42.2	1.6 (1.1, 4.4)	U 1.9 (1.4, 2.6)	2.0 (1.2, 4.9)	U 2.8 (1.9, 4.2)
				SA 1.5 (1.1, 2.0)		SA 2.2 (1.6, 3.2)

Abbreviations: CI=confidence interval; IQR=interquartile range (25th, 75th percentiles); SA=smoking adjusted; U=unadjusted.

Means and their 95% CI calculated from a random effects meta-analysis.

*I*^2^ percentages (% of variability this is due to between-study difference) (heterogeneity *P*-value) for crocidolite, chrysotile+crocidolite, chrysotile, amosite and mixed fibres, respectively, are: for unadjusted *R*_1_: 29% (*P*=0.22), 85% (*P*<0.01), 53% (*P*=0.007), 0% (*P*=0.39), 94% (*P*<0.001); for *R*_2_: 52% (*P*=0.06), 81% (*P*<0.001), 74% (*P*<0.001), 0% (*P*=0.68), 97% (*P*<0.001).

**Table 4:** Estimates of population attributable fractions (%) ranges of lung cancer in men due to exposure to mixed asbestos and crocidolite asbestos fibres

					**Population attributable fraction of lung cancer due to asbestos, estimated for**		
	**No. of deaths^a^**		**Mesothelioma deaths**		**Mixed asbestos fibres**		**Crocidolite**
**Country**	**Lung cancer**	**Mesothelioma**	**Per 1000 all-cause deaths**	**% Of lung cancer deaths^b^**	***R*_1_ 1.5^c^–1.9**	***R*_2_=2.2^c^–2.8**	***R*_1_=0.6^c^, *R*_2_=1.2^c^**
Australia	12 768	1156	7.8	9.1	13.6–17.2	14.7–18.0	5.4, 8.6
UK	90 347	7362	6.9	8.1	12.2–15.5	13.2–16.2	4.9, 7.7
New Zealand	3192	238	5.8	7.5	11.2–14.2	11.3–13.9	4.5, 6.5
Sweden	8386	491	3.2	5.9	8.8–11.1	6.5–8.1	3.5, 3.7
Netherlands	29 604	1629	6.2	5.5	8.3–10.5	12.0–14.8	3.3, 6.9
South Africa	2995	133	0.8	4.4	6.7–8.4	1.8–2.3	2.7, 1.0
Iceland	274	12	3.7	4.4	6.6–8.3	7.5–9.3	2.6, 4.2
Norway	5506	216	2.9	3.9	5.9–7.5	6.1–7.6	2.4, 3.4
Finland	6557	252	2.7	3.8	5.8–7.3	5.5–6.9	2.3, 3.1
Malta	528	20	3.3	3.8	5.7–7.2	6.8–8.4	2.3, 3.8
Denmark	1810	55	2.6	3.0	4.6–5.7	5.4–6.8	1.8, 3.0
Italy	24 126	729	3.4	3.0	4.5–5.7	7.0–8.7	1.8, 3.9
Germany	136 953	4102	2.7	3.0	4.5–5.7	5.5–6.9	1.8, 3.1
France	99 626	2900	2.9	2.9	4.4–5.5	5.9–7.4	1.7, 3.3
Luxembourg	690	16	2.3	2.3	3.5–4.4	4.8–6.0	1.4, 2.7
USA	413 529	8802	2.0	2.1	3.2–4.0	4.3–5.4	1.3, 2.4
Austria	8895	187	1.7	2.1	3.2–4.0	3.7–4.6	1.3, 2.0
Chile	6464	117	0.7	1.8	2.7–3.4	1.5–1.9	1.1, 0.8
Japan	185 988	3010	1.4	1.6	2.4–3.1	3.1–3.9	1.0, 1.7
Spain	76 606	978	1.4	1.3	1.9–2.4	2.9–3.6	0.8, 1.6

a Based on the number of mesothelioma deaths in 1000 deaths, men aged 40–84, years 2001–2005, for countries using ICD 10 (WHO mortality data).

b Note that if there were one asbestos-related lung cancer for every mesothelioma death, this percentage would be the PAF (%) of lung cancer due to asbestos. The ratio and thus PAFs are likely to be higher.

c Corrected for confounding by smoking of up to 20% higher cohort smoking rates compared with the general population.
