# Supplementary material for: Estimating the asbestos-related lung cancer burden from mesothelioma mortality
Source: Br J Cancer. 2012 Jan 10;106(3):575–84. doi: 10.1038/bjc.2011.563 (PMC3273352; doi:10.1038/bjc.2011.563)
Supplement: Supplementary Table 1 [file bjc2011563x1.doc]

**Supplementary Table 1: Asbestos cohort studies providing estimates of lung cancer and mesothelioma mortality**, by asbestos type

| **Est-imate no.** | **Cohort description [reference]** | **N** | **% male** | **Exposure period** | **Follow-up period** | **Evidence** | **Total deaths (O_ALL_)** | **Lung Cancer**  **Mortality** | | | | |  | **Mesothelioma Mortality** | |  | **Ratio Estimates** | |
| --- | --- | --- | --- | --- | --- | --- | --- | --- | --- | --- | --- | --- | --- | --- | --- | --- | --- | --- |
|  |  |  |  |  |  |  |  | **Observed no. (O_LC_)** | **Expected no. (E_LC_)** | **SMR** | **Excess no.** | **Excess relative risk (%)** |  | **Observed (O_M_)** | **No. per 1000 non-asbestos deaths** |  | **R_1_** | **R_2_** |
| **Crocidolite** | | | | | | | | | | | | | | | | | | |
| **1** | Canadian gas mask canisters (McDonald and McDonald, 1978) | 199 | NK | 1939-42 | to 1975 | NS | 55 | 7 | 2·4 | 2·92 | 4·6 | 192 |  | 9 | 217·4 |  | 0·5 | 0·9 |
| **2** | Nottingham gas masks, UK (Jones et al., 1980) | 951 | 0 | 1938-45 | to 1978 | MR | 166 | 12* | 6·3 | 1·90 | 5·7 | 90 |  | 17 | 118·6 |  | 0·3 | 0·8 |
| **3** | Leyland gas masks, UK. Crocidolite with some chrysotile. (Aches*on et a*l., 1982) | 757 | 0 | 1930s-60s | 1951-80 | DC | 219 | 13 | 6·2 | 2·10 | 6·8 | 110 |  | 5 | 22·8 |  | 1·4 | 4·6 |
| **4** | South African crocidolite mines (Sluis-Crem*er et a*l., 1992) | 3430 | 100 | 1925- | 1946-80 | BE | 423 | 27 | 13·3 | 2·03 | 13·7 | 103 |  | 20 | 51·4 |  | 0·7 | 2·0 |
| **5** | Tuscany rail construction Italy (Battis*ta et a*l., 1999) | 734 | 100 | 1945-69 | to 1997 | BE | 199 | 26 | 21·0 | 1·24 | 5·0 | 24 |  | 5 | 26·5 |  | 1·00 | 0·9 |
| **6** | Wittenoom mine/mill, Australia (Musk et al., 2008) | 6943 | 100 | 1943-66 | to 2000 | DC | 2408 | 281 | 108·0 | 2·60 | 173·0 | 160 |  | 222 | 110·3 |  | 0·8 | 1·5 |
| **Chrysotile and crocidolite** | | | | | | | | | | | | | | | | | | |
| **7-** | Rochdale textile workers, UK. Chrysotile, 5% crocidolite during 1932-68. (Peto et al., 1985)  Men, 20+ years employment starting <1933 | 145 | 100 | <1933 | to 1983 | DC | 123 | 20 | 5·6 | 3·61 | 14·4 | 261 |  | 7 | 68·9 |  | 2·1 | 3·7 |
| **8-** | Women, 10+ years employment after 1933 | 283 | 0 | 1933- |  |  | 49 | 4 | 1·9 | 2·11 | 2·1 | 111 |  | 0 | 0 |  | No mesothelioma | |
| **9-** | Principle cohort, men first employed > 1933 | 3211 | 100 | 1932-6 |  |  | 1113 | 132 | 100·5 | 1·31 | 31·6 | 31 |  | 11 | 10·3 |  | 2·9 | 3·1 |
| **10** | Cement, New Orleans Plant 2, US. Chrysotile and crocidolite. (Hugh*es et a*l., 1987) | 3594 | 100 | 1937-69 | to 1981 | DC | 874 | 107 | 74·3 | 1·44 | 32·7 | 44 |  | 4 | 4·8 |  | 8·2 | 9·2 |
| **11** | Vocklabruck cement workers, Austria. Chrysotile and crocidolite. (Neuberger and Kundi, 1990) | 2816 | both | 1950-81 | to 1987 | BE | 540 | 49 | 47·0 | 1·04 | 2·0 | 4 |  | 4 | 7·5 |  | 0·5 | 0·6 |
| **12** | Ferodo friction factory, UK. Predominantly chrysotile, crocidolite use for a short period (Berry, 1994) | 13450 | 68 | 1920-60 | to 1986 | DC | 2577 | 241 | 243·4 | 0·99 | -2·4 | -1 |  | 13 | 5·1 |  | -0·2 | -0·2 |
| **13** | North Israel cement workers, 90% chrysotile, 10% crocidolite (Tulchins*ky et a*l., 1999) | 3057 | 100 | 1953- | to 1992 | INC | *-* | 34 | 27 | 1·26 | 7·0 | 26 |  | 21 | - |  | 0·3 | *-* |
| **14** | Norwegian asbestos cement factory. Predominantly chrysotile, 8% amphiboles (Ulvest*ad et a*l., 2002) | 545 | 100 | 1942-76 | 1953-99 | INC | - | 33 | 10·6 | 3·1 | 22·4 | 210 |  | 18 ^a^ | - |  | 1·2 | - |
| **Chrysotile (pure or predominantly)** | | | | | | | | | | | | | | | | | | |
| **15** | Blackburn gas masks, UK. Pure chrysotile. (Aches*on et a*l., 1982) | 570 | 0 | 1930s-60s | 1951-80 | DC | 177 | 6 | 4·8 | 1·25 | 1·2 | 25 |  | 1 | 5·7 |  | 1·2 | 4·4 |
| **16** | Wales cement workers. After 1936 - chrysotile only. Before 1936 – some crocidolite used. (Thomas et al., 1982) | 1970 | 100 | 1936-77 | to 1977 | DC | 351 | 22 | 25·8 | 0·85 | -3·8 | -15 |  | 2^c^ | 5·7 |  | -1·9 | -2·6 |
| **17** | Connecticut friction industry, US. Pure chrysotile.^d^ (McDonald et al., 1984) | 3531 | 100 | 1938-58 | to 1977 | DC | 803 | 73 | 49·1 | 1·49 | 23·9 | 49 |  | 0 | 0 |  | No mesothelioma | |
| **18** | Swedish cement workers. Chrysotile predominantly, <1% amosite/crocidolite during short periods. (Ohlson and Hogstedt, 1985) | 1176 | 100 | 1943-76 | to 1982 | DC | 220 | 11 | 9 | 1·22 | 2·0 | 22 |  | 0 | 0 |  | No mesothelioma | |
| **19** | Tamworth cement workers, UK. Chrysotile, with amosite used during 4 months out of 42 years(Gardn*er et a*l., 1986) | 2167 | 70 | 1941-83 | 1941-84 | DC | 486 | 41 | 42·4 | 0·97 | -1·4 | -3 |  | 1 | 2·1 |  | -1·4 | -1·6 |
| **20** | Balangero mine, Italy. Pure chrysotile. (Piolatto et al., 1990) | 1058 | 100 | 1946-87 | to 1987 | DC | 427 | 22 | 19·9 | 1·11 | 2·1 | 11 |  | 2^a^ | 4·7 |  | 1·1 | 2·2 |
| **21**  **22**  **23**  **24** | Quebec, Canada: Quebec asbestos factory, chrysotile(Lidde*ll et a*l., 1997)  Thetford company 3, chrysotile and tremolite  Thetford company 4, chrysotile + tremolite  Quebec asbestos mine/mill, chrysotile | 792  4732  368  4503 | 100  100  100  100 | 1904-  1904-  1904-  1904- | to 1992  to 1992  to 1992  to 1992 | DC | 508  3080  267  2924 | 49  280  25  253 | 36·6  193·1  15·2  196·1 | 1·34  1·45  1·65  1·29 | 12·4  86·9  9·8  56·9 | 34  45  65  29 |  | 5  21  2  8 | 9·4  6·3  6·8  2·5 |  | 2·5  4·1  4·9  7·1 | 3·6  7·2  9·5  11·8 |
| **25** | Chongqin asbestos workers, China. Pure chrysotile. May have contained tremolite. (Yano et al., 2001) | 515 | 100 | 1960- | 1972-96 | MR | 132 | 22 | 3·31 | 6·64 | 18·7 | 564 |  | 2 | 18·0 |  | 9·3 | 31·4 |
| **26** | South Carolina textile workers, US. Chrysotile with <0·01% crocidolite(He*in et a*l., 2007) | 3072 | 59 | 1916-77 | to 2001 | DC | 1961 | 198 | 101·5 | 1·95 | 96·5 | 95 |  | 3 | 1·6 |  | 32·2 | 58·9 |
| **27** | North Carolina textile plants, US. Predominantly chrysotile, with some amosite in plant 3 (Loomis et al., 2009) | 5770 | 69 | 1950-73 | to 2003 | DC | 2583 | 277 | 141·7 | 1·96 | 135·3 | 96 |  | 8 ^b^ | 3·3 |  | 16·9 | 29·1 |
| **28** | Greece asbestos cement factory. Chrysotile with 0·5% amphibole contamination (Sichletidis et al., 2009) | 317 | 100 | 1968- | to 2006 | DC/ MR | 52 | 16 | 9·4 | 1·71 | 6·6 | 71 |  | 0 | 0 |  | No mesothelioma | |
| **29** | Chrysotile miners, China (Wang et al., 2011a) | 1080 | 100 | 1958- | 1981-2006 | MR | 343 | 50 | 10.9 | 4.61 | 39.1 | 361 |  | 0 | 0 |  | No mesothelioma | |
| **30** | Chrysotile textile workers, China (Wang et al., 2011b) Medium/high exposure groups compared to low exposed group | 461 | 100 | 1958- | 1972-2008 | PR/DC | 207 | 46 | 25.1 | 1.83 | 20.9 | 83 |  | 2 | 10.9 |  | 10.4 | 7.6 |
| **Amosite** | | | | | | | | | | | | | | | | | | |
| **31** | Uxbridge amosite insulation, UK (Acheson et al., 1984) | 4820 | 100 | 1947-79 | to 1980 | DC | 422 | 57 | 29·1 | 1·96 | 27·9 | 96 |  | 5 | 12·9 |  | 5·6 | 7·5 |
| **32** | Paterson amosite asbestos factory, New Jersey US (Seidman et al., 1986) | 820 | 100 | 1941-54 | 1935- | BE | 593 | 98 | 20·5 | 4·78 | 77·5 | 378 |  | 17 | 34·1 |  | 4·6 | 11·1 |
| 33 | South African amosite mines (Sluis-Crem*er et a*l., 1992) | 3212 | 100 | 1925- | 1946-80 | BE | 648 | 26 | 18·8 | 1·38 | 7·2 | 38 |  | 4 | 6·3 |  | 1·8 | 6·1 |
| 34 | Tyler pipe insulation, amosite, Texas, US (Levin et al., 1998) | 1130 | 100 | 1954-72 | to 1986 | DC | 222 | 35 | 12·6 | 2·78 | 22·4 | 178 |  | 6 | 31·0 |  | 3·7 | 5·7 |
| **Anthophyllite and vermiculite** | | | | | | | | | | | | | | | | | | |
| **35** | Finnish anthophyllite mines, Paakkila, Maljasalmi.  (Meurm*an et a*l., 1994) | 903 | 82 | 1953-67 | 1953-91 | CR, INC | *-* | 77 | 26·9 | 2·86 | 50·1 | 186 |  | 4 | *-* |  | 12·5 | *-* |
| **36** | Vermiculite mine, Libby Montana, US (contains tremolite) (Sullivan, 2007) | 1672 | 96 | 1935- | 1960-2001 | DC | 711 | 89 | 52·6 | 1·69 | 36·4 | 69 |  | 6* | 9·0 |  | 6·1 | 7·7 |
| **Mixed (or unspecified) asbestos types** | | | | | | | | | | | | | | | | | | |
| **37** | Belgian asbestos-cement factory (Lacquet et al., 1980) | 1973 | 100 |  | 1963-77 |  | 201 | 17 | 17·2 | 0·99 | -0·2 | -1 |  | 1 | 5·0 |  | -0·2 | -0·3 |
| **38** | Devonport dockyard, UK (Rossiter and Coles, 1980) | 6292 | 100 | various | to 1978 |  | 1043 | 84 | 100·3 | 0·84 | -16·3 | -16 |  | 31 | 30·1 |  | -0·5 | -0·5 |
| **39** | Danish asbestos-cement factory (Clemmesen and Hjalgrim-Jensen, 1981) | 6372 | 100 | 1943- | to 1976 | INC | *-* | 47 | 27·3 | 1·72 | 19·7 | 72 |  | 3 | - |  | 6·6 | *-* |
| **40** | Pennsylvania textile, US. Mostly chrysotile, some amosite, small amounts crocidolite (McDonald et al., 1983) | 4137 | 100 | 1938-59 | to 1974 | DC | 1392 | 57 | 54·3 | 1·05 | 2·7 | 5 |  | 13 | 9·4 |  | 0·2 | 0·5 |
| **41** | Ontario cement workers, Canada (Finkelstein, 1984) | 535 | 100 | 1950s-70s | to 1977 | DC | 108 | 26 | 5·3 | 4·89 | 20·7 | 389 |  | 17 | 214·8 |  | 1·2 | 1·6 |
| **42** | Swedish rail maintenance (Ohlson et al., 1984) | 3297 | 100 | 1939-80 | 1951-80 | DC | 925 | 37 | 31·8 | 1·16 | 5·2 | 16 |  | 5 | 5·5 |  | 1·0 | 3·0 |
| 43 | Pearl Harbour naval shipyard, US (Kolonel et al., 1985) | 5191 | 100 | WWII | to 1979 | DC | 668 | 122 | 112·2 | 1·09 | 9·8 | 9 |  | 8 | 12·3 |  | 1·2 | 0·7 |
| 44 | Paray-Le-Monial cement factory, France (Alies-Patin and Valleron, 1985) | 1506 | 100 | 1940+ | to 1982 |  | 206 | 12 | 5·5 | 2·18 | 6·5 | 118 |  | 4 | 20·5 |  | 1·6 | 5·8 |
| **45**  **46** | German asbestos workers (Woitowitz et al., 1986)  (a) exposed before 1972 only  (b) exposed before and after 1972 | 655  3070 | NS | < 1972 <>1972 | to 1982 | DC | 71  185 | 12  26 | 2·60  15·29 | 4·62  1·70 | 9·4  10·7 | 362  70 |  | 6  6 | 107·9  35·7 |  | 1·6  1·8 | 3·6  2·0 |
| **47** | Gothenburg shipyard, Sweden(Sanden and Jarvholm, 1987) | 3787 | 100 | to 1972 | 1978-83 | INC | *-* | 11 | 9·8 | 1·12 | 1·2 | 12 |  | 4 | - |  | 0·3 | *-* |
| **48** | Mixed industry, Johns Manville retirees, US (Enterli*ne et a*l., 1987) | 1074 | 100 | 1941-67 | to 1980 | DC | 944 | 77 | 28·44 | 2·71 | 48·6 | 171 |  | 8 | 9·0 |  | 6·1 | 20·2 |
| **49** | Cement, New Orleans Plant 1, US. Primarily chrysotile, irregular use of amosite and crocidolite (Hugh*es et a*l., 1987) | 1898 | 100 | 1942-69 | to 1981 | DC | 477 | 47 | 40·2 | 1·17 | 6·8 | 17 |  | 2 | 4·3 |  | 3·4 | 4·0 |
| **50** | Finnish shipyard workers (Tola et al., 1988) | 7775 | 100 | 1945-60 | to 1981 | INC | *-* | 227 | 192·1 | 1·18 | 34·9 | 18 |  | 1 | - |  | 34·9 | *-* |
| **51** | Southern Sweden cement workers (Alb*in et a*l., 1990) | 1465 | 100 | 1907-77 | to 1986 | BE | 592 | 35 | 19·4 | 1·80 | 15·6 | 80 |  | 13 | 23·1 |  | 1·2 | 3·5 |
| **52** | Asbestos insulation union, US-Canada (Selikoff and Seidman, 1991) | 17800 | 100 | ~1967 | 1967-87 | DC | 3951 | 1168 | 268·7 | 4·35 | 899·3 | 335 |  | 458 | 176·6 |  | 2·0 | 1·9 |
| **53** | Italian rail carriage construction (Menegozzo et al., 1993) | 1534 | 100 | 1970-89 |  | DC | 194 | 28 | 19·31 | 1·45 | 8·7 | 45 |  | 5 | 27·7 |  | 1·7 | 1·6 |
| **54** | Emilia Romagna cement factory, Italy. Chrysotile and 5-50% crocidolite (Giaroli et al., 1994) | 3341 | 100 | 1955-85 | to 1989 | DC | 274 | 33 | 26·6 | 1·24 | 6·4 | 24 |  | 6 | 22·9 |  | 1·1 | 1·1 |
| **55** | Danish Eternit cement factory, 90% chrysotile, amosite and small amounts crocidolite (Raffn et al., 1996) | 7996 | 100 | 1928-84 | 1943-90 | DC | 1305 | 104 | 57·0 | 1·83 | 47·1 | 83 |  | 10 | 8·0 |  | 4·7 | 10·3 |
| **56**  **57**  **58**  **59** | East London asbestos workers UK (Berry et al., 2000) of which: - East London laggers  - East London men  - East London women | 5100  1400  3000  700 | 100  100  0 | 1933- | to 1980 | DC | 1237  -  -  - | 233  38  157  37 | 77·0  10·4  61·6  5·0 | 3·03  3·67  2·55  7·46 | 156·0  27·6  95·4  32·0 | 203  267  155  646 |  | 100  13  60  25 | 101·9  -  -  - |  | 1·6  2·1  1·6  1·3 | 2·0  -  -  - |
| **60** | Genoa shipyard, Italy (Punto*ni et a*l., 2001) | 3984 | 100 | 1960-81 | to 1996 | DC | 2376 | 298 | 168·7 | 1·77 | 129·3 | 77 |  | 60 | 27·4 |  | 2·2 | 2·8 |
| **61** | Lithuania cement, Naujoji Akmeme Lithuania (Smailyte et al., 2004) | 2498 | 69 | 1956-2000 | 1978-2000 | DC | 450 | 38 | 27·1 | 1·40 | 10·9 | 40 |  | 1 | 2·3 |  | 10·9 | 17·5 |
| **62** | Ontario pipe trade, Canada (Finkelstein and Verma, 2004) | 25285 | 100 | > 1949 | age 85 | DC | 2876 | 393 | 320·1 | 1·23 | 72·9 | 23 |  | 8 | 2·9 |  | 9·1 | 8·0 |
| **63** | US Coast Guard shipyard workers (Krst*ev et a*l., 2007) | 4702 | 94 | 1950-64 | to 2001 | DC | 3331 | 314 | 248·3 | 1·26 | 65·7 | 26 |  | 6 | 1·8 |  | 11·0 | 14·4 |
| **64** | Grugliasco textile, Italy. Mixed asbestos including crocidolite (Pira et al., 2007) | 1966 | 45 | 1946-84 | to 2004 | DC | 730 | 109 | 35·1 | 3·11 | 73·9 | 211 |  | 68 | 115·6 |  | 1·1 | 1·8 |
| **65** | Breda Pistoia railway rolling factory, Italy (Gasparrini et al., 2008) | 1146 | 100 | 1956-79 | 1960-2004 | DC | 1080 | 132 | 97·1 | 1·36 | 34·9 | 36 |  | 16 | 15·5 |  | 2·2 | 2·3 |
| **66**  **67** | Great Britain Asbestos Survey (Harding, 2010) - men  - women | 94403  4509 | 100  0 | 1920s- | 1971-2005 | CR | 14677  880 | 1802  84 | 963·1  41·9 | 1.28^e^  1.29^e^ | 397·5  18·9 | 28·3  29·1 |  | 631  18 | 46·2  21·4 |  | 0·6  1·1 | 0·6  1·4 |
| **68** | Japanese shipyard (laggers and boiler repairers) (Tomio*ka et a*l., 2011) | 249 | 100 | 1947-79 | 1947-2007 | DC | 158 | 15 | 7.6 | 1.97 | 7.4 | 97 |  | 1 | 6.7 |  | 7.4 | 14.5 |

INC: Incidence data only, no mortality data. NS, not specified; DC, death certificate; CR, cancer registry or mesothelioma register; BE, best evidence; PR, employer’s records;

^a^ Pleural mesothelioma only.

^b^ 4 cancers of pleura and 4 mesothelioma (latter only available from 1999 with ICD10). The 4 mesotheliomas were in workers who had been employed in plant 4 where there was no record of amphibole use. 3 pleural cancers were in plant 3, but these workers had not worked in the insulation section of plant 3 where amosite was used.

^C^ The 2 mesotheliomas were in men who were employed prior to 1936 when exposure to crocidolite was likely.

^d^ Deaths 20 years after first employment only.

^e^ Proportional mortality ratio reported rather than SMR.
